# Effectiveness of internet-based nursing interventions for the treatment of patients with periodontitis

**DOI:** 10.1186/s12903-024-04147-3

**Published:** 2024-03-26

**Authors:** Lihua Liang, Yiwen Sheng, Mengli Wang, Xiaojun Li, Zijian Cheng

**Affiliations:** 1 Stomatology Hospital, School of Stomatology, Zhejiang University School of Medicine, Zhejiang Provincial Clinical Research Center for Oral Diseases, Hangzhou, Zhejiang China; 2 Key Laboratory of Oral Biomedical Research of Zhejiang Province, Cancer Center of Zhejiang University, Hangzhou, Zhejiang China

**Keywords:** Internet, Nursing interventions, Periodontitis, Oral hygiene instruction, Dental plaque control

## Abstract

**Background:**

Periodontitis, one of the most common oral diseases, is a chronic inflammatory condition occur in response to bacterial plaque biofilms. Plaque control and oral hygiene instructions are the most widely used and effective nonsurgical treatment for periodontitis, which is based on a partnership between patient and clinician and requires a life-long commitment. The objective of this study was to analyze the effectiveness of internet-based nursing interventions for the treatment of patients with periodontitis. The findings from this study may help to enhance the therapeutic outcomes for patients with periodontitis.

**Methods:**

A total of 80 patients with periodontitis treated in Zhejiang Province Stomatology hospital from December 2021 to January 2023 were randomly selected and divided into control group and intervention group with 40 cases each. The control group was given routine oral health guidance and the intervention group received internet based nursing intervention. The periodontal pocket depth, percentage of periodontal pocket depth (PD) ≥ 4 mm, bleeding on probing (BOP)%, and self-efficacy scale for oral health care (SESS) were assessed and compared at four time points: initial visit, 6-8-weeks follow-up, 3-months follow-up, and 6-months follow-up.

**Results:**

There was no significant difference between the two groups in terms of age, gender, initial visit PD, initial visit PD ≥ 4 mm (%), initial visit BOP (%), and initial visit SESS (*P* > 0.05). The intervention group showed a significantly decreased percentage of PD ≥ 4 mm at 6–8 weeks and 6-months follow-up compared to the control group (*P* < 0.05). The PD, BOP%, and SESS scores of the intervention group were significantly better than those of the control group at 6-months follow-up (*P* < 0.05). There was no statistically significant difference in patient satisfaction between the two groups.

**Conclusions:**

This study confirmed that the internet-based nursing intervention in conjunction with periodontal treatment was able to improve the periodontal pocket depth, gingival bleeding and the level of self-efficacy of patients, suggesting that it is necessary to carry out the extended oral hygiene instructions via internet-based platforms for the patients in clinical practice.

**Supplementary Information:**

The online version contains supplementary material available at 10.1186/s12903-024-04147-3.

## Background

Periodontitis, the most common oral disease, is a chronic inflammatory condition occur in response to bacterial plaque biofilms, characterized by the destruction of soft and hard tooth-supporting tissue and ultimately leading to tooth loss [1]. Severe periodontitis is ranked as the sixth most common disease in the world, with an incidence of 11.2% among the global adult population [2]. It is now generally accepted that periodontitis is initiated by the colonization of dental plaque by a set of pathogenic bacteria, referred to as the “red complex”, which are well equipped with a broad array of virulence factors and strongly associated with the disease [3].

The key to the treatment of periodontitis is to remove the plaque and bacterial products planted on the root surface, remove the diseased periodontal tissues, stop the progress of the disease, and promote the regeneration of periodontal tissues. Nonsurgical periodontal treatment for plaque control is the most widely used and effective treatment for periodontitis [4, 5]. Management of periodontal health is a partnership between patient and clinician and requires a life-long commitment. Dentists who work with individuals affected by periodontal disease face the challenge of promoting compliance with oral hygiene instructions [5]. Previous studies have demonstrated that it is difficult for people to master appropriate self-care methods and skills [6] and it has been suggested that use of goal setting, self-monitoring and planning are effective interventions for improving oral hygiene-related behavior in patients with periodontitis [7, 8]. Verbal and written advice are the two most commonly employed methods for promoting oral health. However, research data indicate that these approaches may not significantly impact the development of diseases [9].

In China, dental nurses have the same knowledge as other medical nurses so that they can work in an oral surgical ward nursing in-patients as well in dental clinics [10] and play an important role in oral care interventions [11].The widespread popularity of smartphones and tablets has created a convenient and accessible platform for nursing experts to guide individuals with chronic diseases anytime and anywhere [12, 13]. At present, the WeChat platform is used in the inland of China for continuous nursing of patients with chronic disease [14]. This new pathway allows for continuous support and guidance, ensuring that individuals can receive assistance and advice at their convenience. Recent systematic reviews have demonstrated that information-based technologies used as an adjunct component in managing periodontal health [13, 15]. However, little is known about the role of nursing interventions and their effect on the treatment of periodontitis.

Therefore, the main aim of this study was to investigate the effectiveness of internet-based nursing interventions for patients with periodontitis. The findings from this study should provide vital information for the clinical practice to enhance therapeutic outcomes for patients with periodontitis.

## Methods

This study was conducted in accordance with the Helsinki Declaration as revised in 2013. The study protocol was approved by the Stomatology Research Ethics Committee of the Affiliated Stomatology Hospital (No. 2021-122R-X1) of Zhejiang University School of Medicine. Informed consent was obtained from all participants.

### Study population

**Fig. 1:**
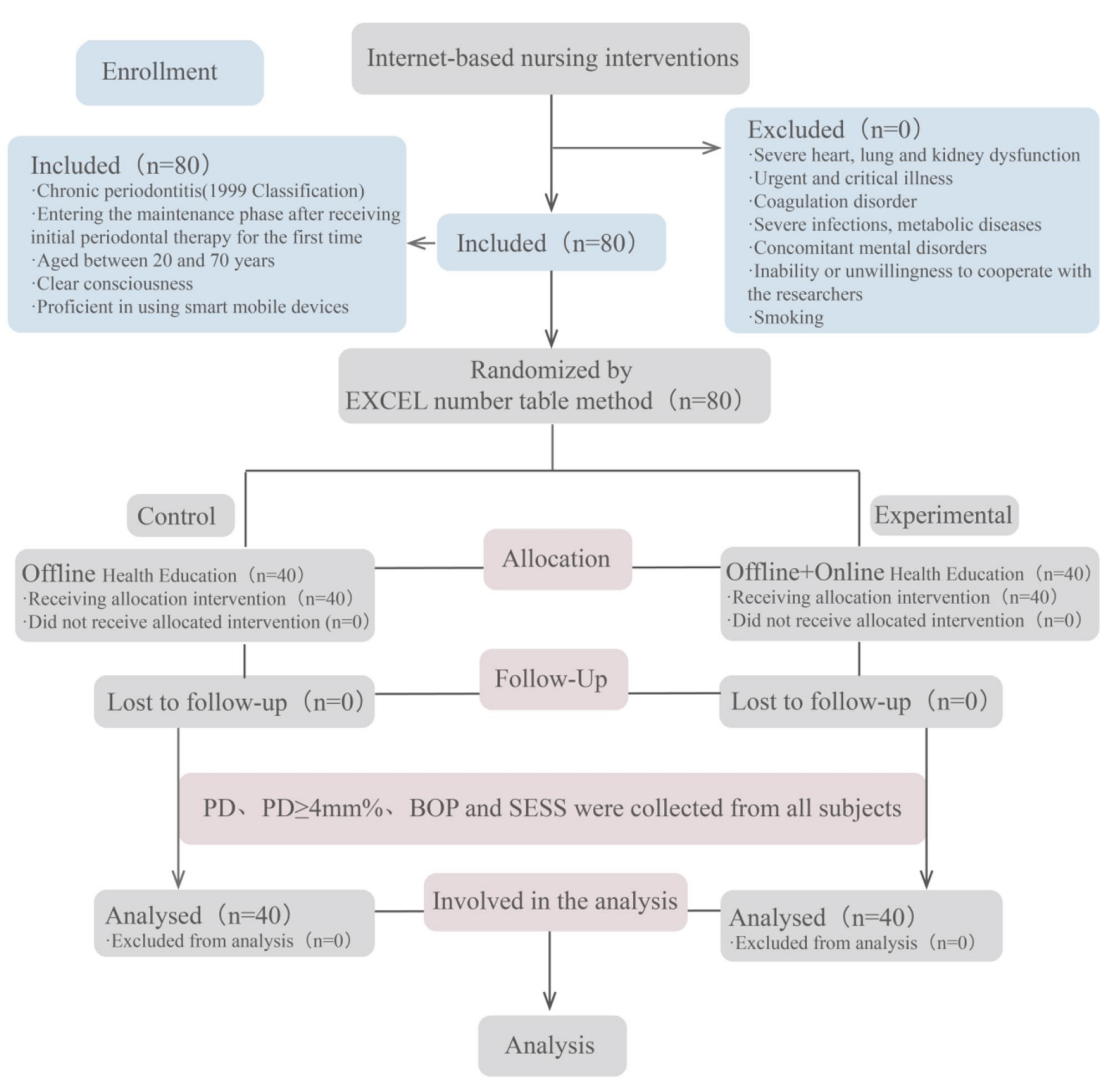
Research process

A total of 80 patients were enrolled in this study and were randomly divided into control and intervention groups using the Excel spreadsheet method, with 40 patients in each group (Fig. 1).The study subjects were patients with chronic periodontitis who visited the department of Periodontics at Stomatology Hospital affiliated to Zhejiang University in China between December 2021 to January 2023. Socio-demographic data were collected including age, gender, employment and education level. Inclusion criteria: (1) chronic periodontitis was determined according to the recent update to the International Symposium on the Classification of Periodontal Diseases [16]; (2) received nonsurgical treatment for periodontitis; (3) aged between 20 and 70 years, with no gender restriction; (4) clear consciousness, without cognitive or behavioral dysfunction; (5) proficient in using smart mobile devices.

Exclusion criteria: (1) severe heart, lung, or kidney dysfunction; (2) acute critical illness; (3) coagulation dysfunction; (4) severe infection or metabolic disease; (5) concomitant mental disorders; (6) inability or unwillingness to cooperate with the researchers. (7) smoking.

### Study design

#### Control group

The control group received routine health care, including: (1) demonstrating the correct BASS tooth brushing method and providing oral hygiene guidance for patients during the hospital visit; (2) implementing oral health education after the treatment by traditional patient leaflets. Patients were instructed on key points from the leaflets to gain specific and detailed oral health knowledge, strengthen their oral health awareness, and guide them in the correct use of dental floss or interdental brushes.

#### Intervention group

In addition to routine health care, the intervention group implemented a health intervention model based on internet, which included: (1) establishing a WeChat group for patients and sending them information about their oral hygiene twice a day and answering patients’ any questions about oral health; (2) displaying videos about the BASS tooth brushing method, dental floss, and interdental brushes on screens in the waiting area for patients to watch and learn while waiting; for patients with severe cases, nurses would accompany or remind them to watch the videos and answer any questions; (3) registering patients’ contact information and providing telephone follow-ups during their revisit periods, supervising and guiding their oral hygiene behavior, and using phone reminders and WeChat group messages to encourage timely visits; for particularly severe cases, increasing the frequency of follow-ups to improve patients’ understanding of periodontal disease; (4) providing one-on-one psychological counseling for patients, communicating with them in a timely manner, patiently answering their questions, and establishing a good doctor-patient relationship while emphasizing the importance of regular periodontal maintenance and encouraging patients to revisit and actively cooperate with treatment, relieving their psychological stress and guiding them in maintaining good personal oral hygiene; (5) generating QR codes for post-treatment health education content, tooth brushing, interdental brush, and dental floss usage, allowing patients to take photos and save them for easy access to educational materials and videos.

### Establishing an internet-based nursing intervention team

The team consisted of 2 periodontal specialists, 1 head nurse of stomatology and 2 nurses. The periodontal specialists and the head nurse of stomatology served as team leaders and give the guidance and advice to other team members. All team members received unified training and were qualified for periodontal examination and providing oral hygiene instructions for the patients with periodontitis. The periodontal probes were positioned parallel to the long axis of the tooth and were always in contact with the tooth using approximately 20–25 g of force. During the clinical data collection process, one of the two periodontal specialists was responsible for chair-side clinical examination. The other was responsible for chair-side supervision and data recording. All nurses received training of unified the speaking skills and the way of using oral hygiene tools (tooth brush, interdental brush, and dental floss) to ensure that patients in the intervention group received the same information.

### Outcome measurements

The evaluation indicators of both groups are measured at four time points: initial visit, 6–8 weeks follow-up, 3 months follow-up, and 6 months follow-up.

#### Periodontal pocket probing depth

Periodontal pocket depth is the distance from the gingival margin to the pocket bottom. Healthy gingival sulcus probing depth does not exceed 2-3 mm. Probing method: (1) hold the probe (UNC15) with a modified pen grip; (2) use the labial surface of the adjacent tooth or the proximal edge as a fulcrum, or use an extraoral fulcrum; (3) apply light probing force, about 20-25 g; (4) the probe should be parallel to the tooth’s long axis when inserted. The probe should be close to the tooth surface, avoiding entry into soft tissue, bypassing calculus, and reaching the pocket bottom until slight resistance is felt at the sulcus bottom; (5) move the probe with a lifting and inserting motion, sequentially probing the periodontal pocket depth of the six points on the labial (buccal) and lingual (palatal) sides of each tooth, taking the average value.

#### Bleeding on probing (BOP)

Use a blunt-tipped periodontal probe (UNC15) to gently probe the pocket bottom or sulcus bottom from the buccal, lingual, proximal, and distal sides. After removing the probe, observe for 10–15 s for bleeding, and record the results. The ratio of bleeding points to all points is the BOP%.

#### Self-efficacy scale for self-care (SESS) score

Self-efficacy level of patients with chronic periodontitis was assessed using the SESS [17]. The scale consists of 15 items, including self-efficacy for regular dental visits, proper tooth brushing, and balanced diet. It uses a Likert 5-point scale, scoring 1–5 points from “completely unconfident” to “very confident.”

#### Satisfaction

Patients’ satisfaction with nursing care was assessed by filling out a designed questionnaire. The questionnaire evaluated the clinic environment, waiting area cleanliness, nursing staff service attitude, health education content, nursing staff answering questions, respecting privacy, service instructions, waiting time, and professional ethics of medical staff. The results divided into three categories that included satisfied, general, and need improvement, with scores of 3 − 1 points, respectively. The questionnaire also asked about whether willing to introduce other patients to the hospital. is scored as or, with the values ranging from 2 (willing) to 1 (unwilling). The total evaluation score ranges from 10 to 29 points, with higher scores indicating higher patient satisfaction.

### Statistical analysis

SPSS 26.0 software was used for statistical analysis. Count data are described using frequency and rate, and comparisons between groups are made using the χ2 test. Measurement data are tested for normality using the Shapiro-Wilk test. Normally distributed data are described using mean ± standard deviation. Comparisons between groups for baseline data are made using independent-sample T-tests, and outcome data comparisons are made using analysis of covariance. Skewed data are described using median and quartiles, and comparisons between groups are made using the Mann-Whitney U test. Repeated measures data at multiple time points within groups are compared using the Friedman M test. The test level is α = 0.05.

## Results

### General information

General information includes age and gender. In this study, there are 15 males and 25 females in the control group, aged 23–61 years, with an average age of 40.75 ± 10.24 years; and 12 males and 28 females in the intervention group, aged 23–68 years, with an average age of 40.7 ± 11.24 years. There was no significant difference between the two groups in terms of age, gender, employment, education level, initial visit PD, initial visit PD ≥ 4 mm (%), initial visit BOP (%), and initial visit SESS (*P* > 0.05, Table 1).

**Table 1:** Baseline characteristics

	Control group *n* = 40	Intervention group *n* = 40	T / X^2^/ Z	*P* value
Sex			0.50^2)^	0.48
Male	15	12		
Female	25	28		
Age (Median, QR)	40.00(32.00, 50.00)	37.50(32.00, 52.25)	0.28^3)^	0.78
Employment			0.29^2)^	0.59
Unemployed or enrolled Employed Retired	2 35 3	1 34 5		
Education level			0.05^2)^	0.82
Less than college degree College degree or above	15 25	14 26		
PD (Median, QR)	3.65(3.20, 3.90)	3.55(3.20, 3.90)	0.63^3)^	0.53
PD ≥ 4 mm % (mean ± SD)	40.92 ± 22.58	35.59 ± 18.18	1.16^1)^	0.25
BOP%(Median, QR)	78.50(62.25, 88.00)	77.00(63.00, 83.75)	0.59^3)^	0.56
SESS (Median, QR )	22.00(18.00, 27.00)	20.00(17.00, 29.75)	0.76^3)^	0.45

BOP: Bleeding on probing; PD: probing depth; SESS: self-efficacy scale for oral health care; QR: Quartiles; SD; Standard deviation

^1)^independent-sample t test; ^2)^chi-squared test; ^3)^Mann-Whitney *U* test

### Effect of internet-based nursing interventions on the periodontal treatment

**Fig. 2:**
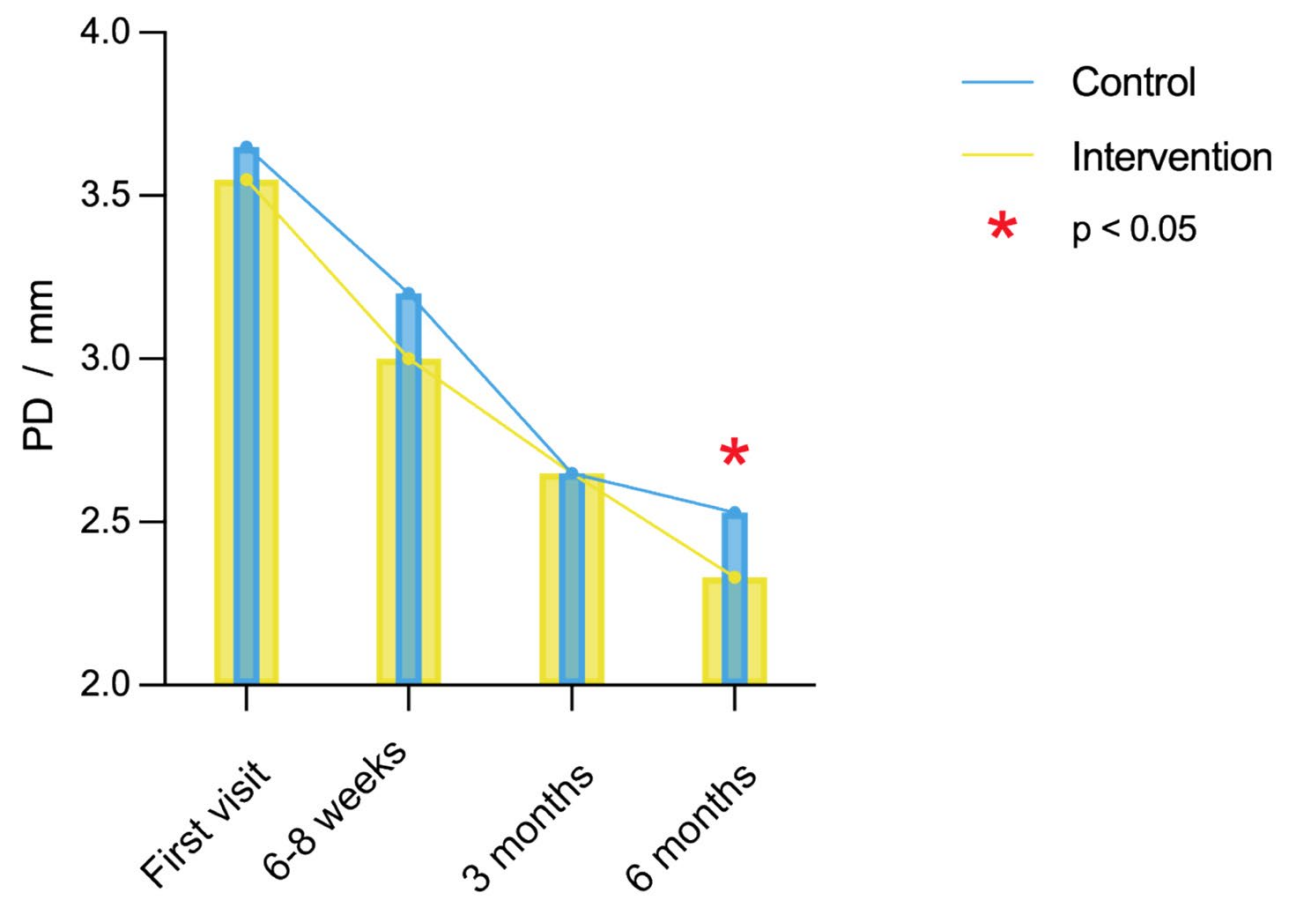
Evaluation of probing depth

Overall, there was a decrease in PD after periodontal treatment at 6–8 weeks, 3 months, and 6-months follow-up in the control group (*x*^*2*^ = 108.37, *P* < 0.001) and the intervention group (*x*^*2*^ = 106.32, *P* < 0.001). There was no significant difference in PD at 6–8 weeks (*Z* = 1.68, *P* = 0.09) and 3-months (*Z* = 0.31, *P* = 0.76) follow-up between intervention group and control group. However, the intervention group showed significantly lower PD at 6-months follow-up (*F* = 2.16, *P* < 0.05, Fig. 2), indicated that internet-based nursing interventions may had a positive effect on the treatment of patients with periodontitis.

**Fig. 3:**
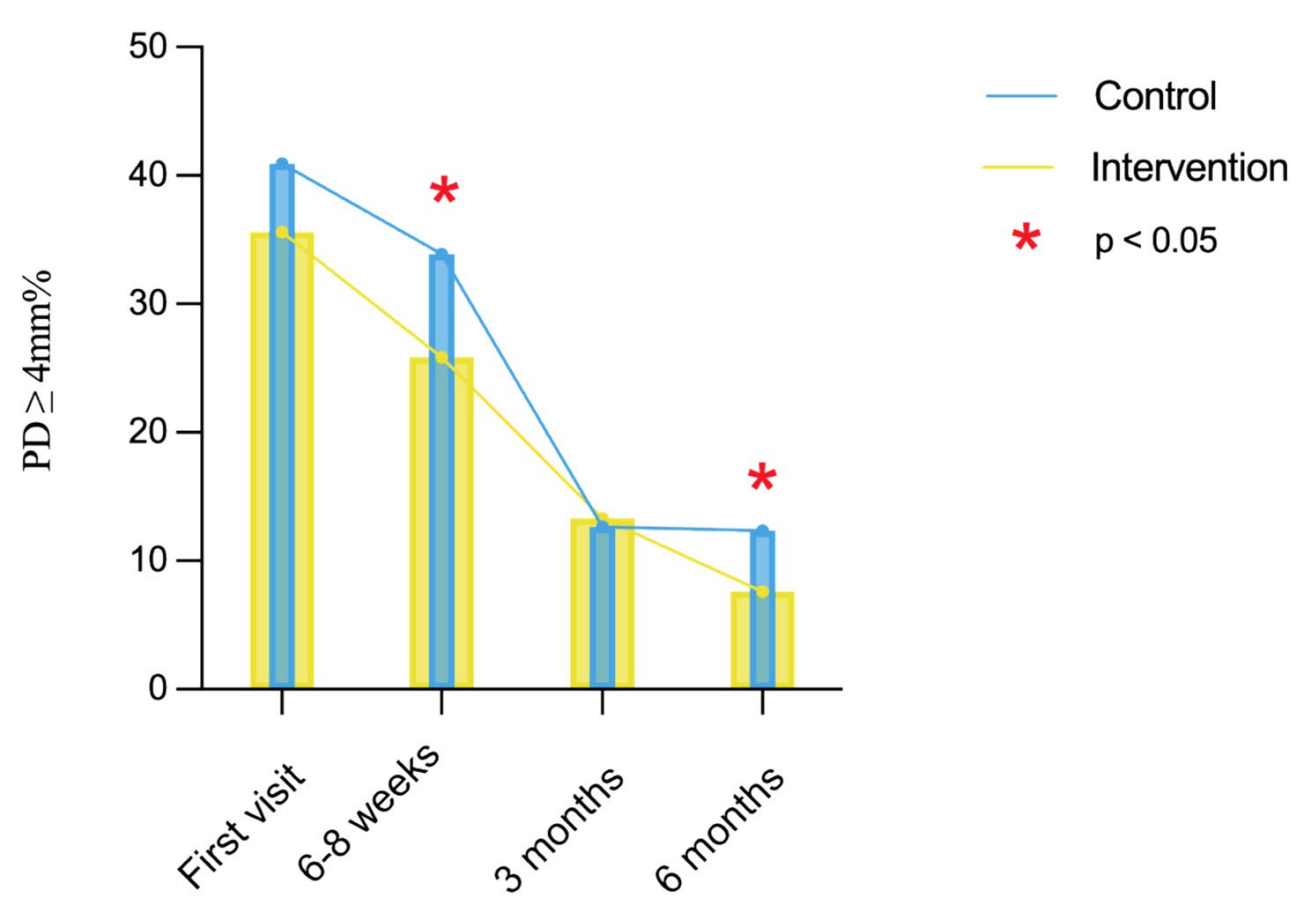
Evaluation of probing depth ≥ 4 mm%

**Fig. 4:**
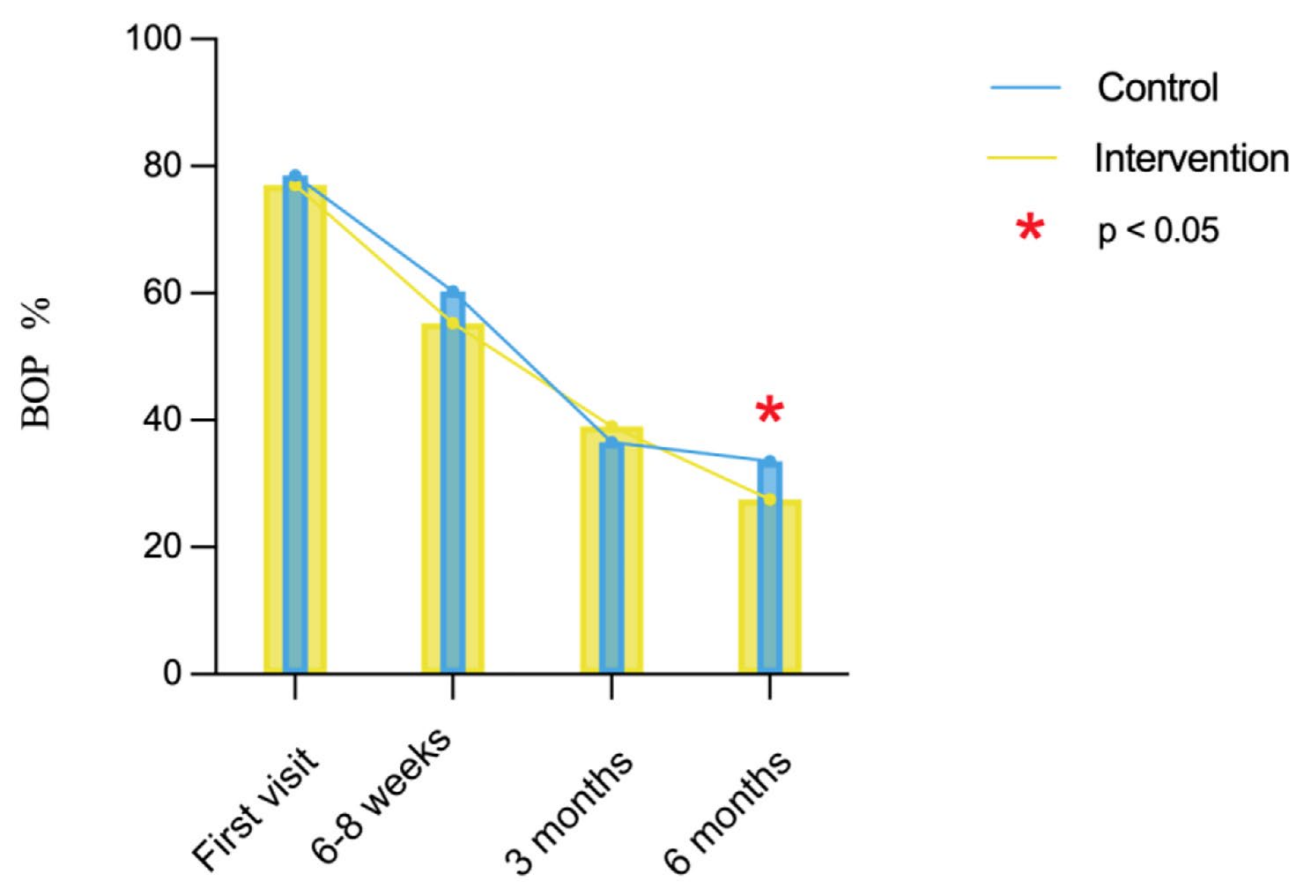
Evaluation of bleeding on probing

Accordingly, the percentage of PD ≥ 4 mm exhibited a similar trend after periodontal treatment and showed a significant decrease in the intervention group, compared with the control group at 6–8 weeks and 6-months re-visit (*F* = 2.87, *P* < 0.05, Fig. 3). Similarly, the percentage of BOP significantly decreased after the treatment in the control group (*x*^*2*^ = 94.97, *P* < 0.001) and the intervention group (*x*^*2*^ = 103.16, *P* < 0.001). No significant difference in BOP% was found between the two groups until 6-months follow-up (*Z* = 2.00, *P* < 0.05, Fig. 4).

### Evaluation of SESS and patient satisfaction

**Fig. 5:**
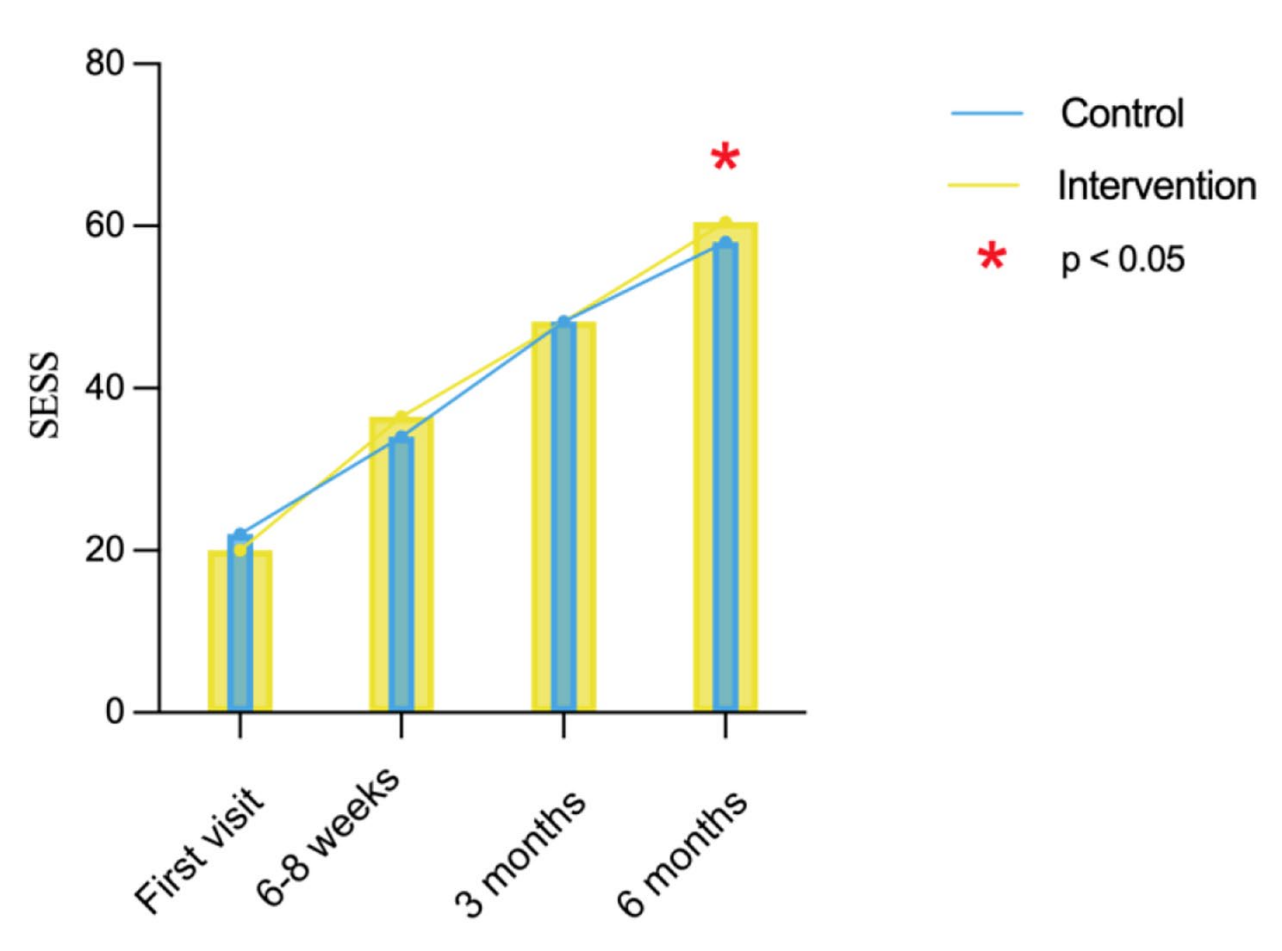
Evaluation of self-efficacy scale for oral health care

Overall, SESS scores of both control group and intervention group showed an increase at 6–8 weeks, 3 months, and 6-months follow-up (control: *x*^*2*^ = 118.82, *P* < 0.001; intervention: *x*^*2*^ = 115.99, *P* < 0.001). When analyzing the intergroup effects between the control group and the intervention group, there was no statistically significant difference in SESS scores at 6-8-weeks follow-up (*Z* = 0.57, *P* = 0.57) and 3-months follow-up (*F* = 0.08, *P* = 0.94). However, there was a statistically significant difference in SESS scores at 6-months follow-up (*F* = 1.07, *P* < 0.05, Fig. 5). There was no statistically significant difference in patient satisfaction between the two groups (*Z* = 0.368, *P* = 0.713, data now shown).

## Discussion

In the traditional model, outpatient patients leave the hospital after completing treatment, have little contact with medical staff, and are difficult to manage. Therefore, it is necessary to construct a holistic, continuous, and extended nursing intervention model for chronic periodontitis patients [18]. This study investigates the effectiveness of internet-based nursing interventions for patients with periodontitis. Results confirmed our hypothesis of a favorable treatment effect of internet-based nursing interventions when compared to the standardized oral hygiene instructions. Our analysis showed that internet-based nursing interventions were able to reduce PD, PD ≥ 4% and BOP% more effectively than the other interventions at 6-months follow-up. Consistent with the findings of a randomized controlled trial conducted by Brand et al. [19], the results indicate that patients in the intervention group experienced more significant improvements in their periodontal health. Internet based nursing interventions can help patients gain a comprehensive understanding of the risk factors associated with periodontal disease and empower them to take control. In the supportive of our results, a randomized controlled trial conducted by Shida et al., showed that a digital device providing real-time visualized brushing instructions may be effective for the removal of dental plaque [20], although the periodontal status were not examined in their study. Similarly, a recent systematic review with meta-analysis assessed the effectiveness of mobile applications and text messages in reducing gingival inflammation. The findings of the review demonstrated that the implementation of mobile health strategies yielded better outcomes when compared to conventional oral hygiene instructions [15]. In comparison to conventional strategies, internet-based nursing interventions offer the advantage of providing structured oral hygiene instructions and facilitating the arrangement of specific and extended educational activities through phone reminders and WeChat group messages. Additionally, they allow for repetition and reinforcement of education between dental appointments, which holds significant potential for improving periodontal treatment outcomes. However, there was no significant difference at 6–8 weeks and 3-months follow-ups between intervention group and control group in present study, although both groups showed better periodontal conditions after treatment. It is reasonable that long-term oral hygiene practices are necessary to achieve the noticeable effects on the periodontal status following the internet-based nursing interventions. This might also be explained by the limitations that presence of confounding factors such as body mass index, alcohol consumption and socio-demographics that were not included in the study.

Previous studies have shown that individuals’ self-efficacy in oral health have an important influence on their compliance with supportive periodontal care. Higher self-efficacy is associated with better compliance to the periodontal treatment and maintenance, which may lead to a better prognosis of periodontitis [21]. In this study, there were no significant differences in the total self-efficacy scores and scores of each dimension between the intervention group and the control group before the intervention (*P* > 0.05). At 6–8 weeks and 3 months’ reexamination, the intervention group had higher total self-efficacy scores compared to the control group. However, the difference was not statistically significant (*P* > 0.05). This may be attributed to the various discomforts experienced by patients due to periodontitis, such as loose or missing teeth, bad breath, gum bleeding, and dental abscesses, which could affect their psychological well-being. Patients tend to reflect on their past behaviors and pay extra attention to their periodontal conditions before and one month after treatment. The most freqluently asked question by the patients was about the influence from the delay or cancellation of the follow-up appointment (supplement Fig. 1). To encourage patients to follow the treatment plan, our team explained the necessity of follow-up appointment and emphasized its role in the management of periodontal health. After 6 months of intervention, there was a statistically significant difference in the total self-efficacy scores between the two groups (*P* < 0.05). In agreement with the results of previous study [22], our study indicates that internet-based nursing intervention are more effective in improving oral self-care efficacy in patients with periodontitis in the long term compared to conventional oral health instructions.

In this study, there was no statistically significant difference in patient satisfaction scores between the intervention group and the control group. This suggests that the nurses’ intensive multi-channel follow-up did not cause unnecessary trouble for the patients and the method used in the internet-based intervention was accepted by the patients. However, it is important to exercise caution when interpreting these findings due to the limited sample of patients from a single Three-A hospital in Zhejiang province. Additionally, most participants in this study were young adults (control group: 40.75 ± 10.24 years; intervention group: 40.7 ± 11.24 years) who are familiar with and come from digital culture, which may have facilitated their access to the internet platform and information [23]. Therefore, the findings of this study may not apply to elder population and future multi-center randomized controlled studies involving a wider range of age groups should be conducted to further investigate the effectiveness of internet-based nursing interventions. Although the two groups were well balanced for age, sex, and smoking status, it is important to note that this study had a relatively small sample size. To obtain a more comprehensive and profound evaluation of the application’s effectiveness, future studies should consider increasing the sample size and extending the intervention period. In addition to the smoking, diabetes mellitus is one of the major risk factors for periodontitis. Individuals with diabetes are more likely to have periodontitis of increased severity when their diabetes is poorly controlled [24]. . The findings of a recent systematic review demonstrated that patients with diabetes and periodontitis had improved periodontal status after nonsurgical periodontal treatment [25]. None of the patients in present study had been diagnosed with diabetes mellitus (data not shown), however, the blood glucose or HbA1c level of the patients were not included in present. It would be interesting to evaluate the effect of ICT-based nursing interventions on the treatment of periodontitis combined with diabetes mellitus by recording the blood glucose or HbA1c levels of the patients.

In addition to periodontal disease, dental caries is also a common oral disease mediated by bacteria and can be prevented by satisfactory dental biofilm control via oral hygiene instruction. Therefore, it would be advisable to implement internet-based nursing interventions for patients with dental caries in the future to enhance disease prevention and treatment outcomes.

## Conclusion

In summary, the results of this study confirmed that the internet-based nursing intervention in conjunction with periodontal treatment was able to improve the self-efficacy of patients and their periodontal status, mainly manifested as the decrease of periodontal pocket depth and gingival bleeding. This study suggested that it is necessary to carry out extended oral hygiene instructions via convenient internet platforms for the patients in clinical practice.

### Electronic supplementary material

Below is the link to the electronic supplementary material.

Supplementary Material 1

## Data Availability

The data of this study are available from the corresponding authors upon reasonable request and with permission of Affiliated Stomatology Hospital of Zhejiang University School of Medicine.

## Abbreviations

BOP: Bleeding on probing
PD: Probing depth
SESS: Self-efficacy scale for oral health care
QR: Quartiles
SD: Standard deviation
