# Supplementary material for: Effectiveness of internet-based nursing interventions for the treatment of patients with periodontitis
Source: BMC Oral Health. 2024 Mar 26;24:386. doi: 10.1186/s12903-024-04147-3 (PMC10964612; doi:10.1186/s12903-024-04147-3)
Supplement: Supplementary file 1 — Supplementary Material 1 [file 12903_2024_4147_MOESM1_ESM.docx]

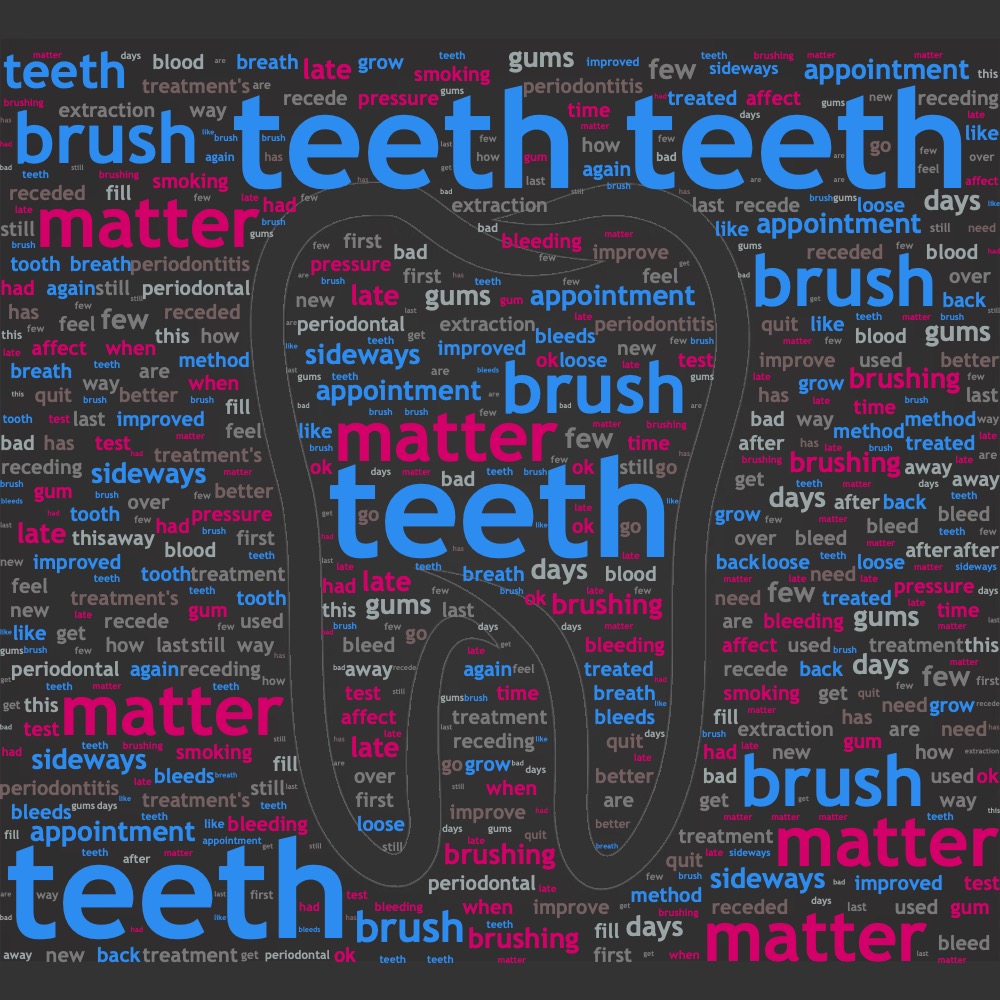


**Supplementary figure 1. Word cloud generated using the conversation history between patients and nurses. The size of the words represents the frequency of occurrence.**
